# Upregulation of Linc00284 Promotes Lung Cancer Progression by Regulating the miR-205-3p/c-Met Axis

**DOI:** 10.3389/fgene.2021.694571

**Published:** 2021-09-20

**Authors:** Wang Sheng, Weixi Guo, Fang Lu, Hongming Liu, Rongmu Xia, Feng Dong

**Affiliations:** ^1^Department of Medical Oncology, Xiamen Key Laboratory of Antitumor Drug Transformation Research, The First Affiliated Hospital of Xiamen University, School of Clinical Medicine, Fujian Medical University, Fuzhou, China; ^2^Department of Thoracic Surgery, The First Affiliated Hospital of Xiamen University, Xiamen, China; ^3^Pulmonary and Critical Care Medicine (PCCM), The First Affiliated Hospital of Xiamen University, Xiamen, China; ^4^School of Medicine, Xiamen University, Xiamen, China; ^5^Department of Radiotherapy, The First Affiliated Hospital of Fujian Medical University, Fuzhou, China

**Keywords:** LINC00284, miR-205-3p, c-Met, lung cancer, ceRNA

## Abstract

Lung cancer (LC) is a malignant tumor with the highest incidence and mortality rates worldwide. Linc00284, a long non-coding RNA, is a newly discovered regulator of LC. This study aimed to explore the role of Linc00284 in LC progression. Gene expression levels were detected by RT-qPCR and/or western blot analysis. Cell migratory and invasive capabilities were measured by wound healing and transwell assays. Subcutaneous xenograft models were constructed to examine tumor growth of LC cells. Data showed that Linc00284 was significantly upregulated in LC tissues compared to adjacent normal lung tissues and predicted poor prognosis in patients with LC. *In vitro*, Linc00284 was highly expressed in LC cells and was mainly localized in the cytoplasm. Mechanistically, Linc00284 directly bound to miR-205-3p, leading to the upregulation of c-Met expression. A significant negative correlation was observed between Linc00284 and miR-205-3p expression levels, and the Linc00284 level was positively correlated with the c-Met expression. Linc00284/miR-205-3p/c-Met regulatory axis promotes LC cell proliferation, migration, and invasion. Furthermore, the *in vivo* results indicated that Linc00284 knockdown markedly suppressed tumor growth. Taken together, these data suggest that Linc00284 facilitates LC progression by targeting the miR-205-3p/c-Met axis, which may be a potential target for LC treatment.

## Introduction

Lung cancer (LC) is the leading cause of cancer-associated mortality worldwide (Siegel et al., 2020). Although previous studies have demonstrated that various factors, including smoking (Schwartz and Cote, 2016; Tang et al., 2019), environmental pollution (Radziszewska et al., 2015), and genetic mutations (Sharma et al., 2007), are involved in the pathogenesis of LC, the molecular mechanisms responsible for LC initiation and progression are not yet fully clarified. Long intergenic non-coding RNAs (lncRNAs) have been shown to play important roles in various pathophysiological processes (Yang et al., 2014; Gao et al., 2018; Lei et al., 2019). LincRNAs belong to long non-coding RNAs (lncRNAs; Deniz and Erman, 2017; Ransohoff et al., 2018). LincRNAs are significantly associated with the development and progression of various cancer types (Chen et al., 2017; Ma et al., 2018). LincRNAs modulate oncogene expressions, promoting the malignant phenotypes of tumors, including LC (Lu et al., 2017; Peng et al., 2018; Wang et al., 2019), gastric cancer (Chen et al., 2018), and colon cancer (Chang et al., 2018).

Non-coding RNA in the aldehyde dehydrogenase 1 A pathway (NRAD1) is known as LINC00284 (Vidovic et al., 2020) with a length of 2,564 bp and is located on chromosome 13q14.11. An abnormally high expression of LINC00284 has been observed in several types of cancer (Xing et al., 2018; Zhao et al., 2018; Ruan and Zhao, 2019; Vidovic et al., 2020; Wang et al., 2020a, b). Research into primary papillary thyroid cancer (PTC) has demonstrated that LINC00284 is tightly associated with the overall survival of patients with PTC (Zhao et al., 2018). Besides, in human gastric cancer, LINC00284 is closely related to overall survival (Xing et al., 2018). Targeting LINC00284 using antisense oligonucleotides has been shown to suppress cell survival and tumor growth in triple-negative breast tumors (Vidovic et al., 2020), and to contribute to reduced angiogenesis in ovarian cancer (Ruan and Zhao, 2019). However, the role of LINC00284 in LC remains unknown. Hence, the present study aimed to investigate the biological function of Linc00284 in LC progression and identify a potential target for LC diagnosis and treatment.

## Materials and Methods

### Human Tissue Samples

This study was approved by the Ethics Committee of The First Affiliated Hospital of Fujian Medical University (Approval no. FMU2018-109-16). Written informed consent was obtained from all patients prior to sample collection. A total of 43 paired LC and adjacent normal tissues were collected from patients enrolled in the present study from The First Affiliated Hospital of Fujian Medical University between July, 2013 and November, 2019. The inclusion criteria used are described below: patients with lung cancer had not received any chemoradiotherapy and/or immunotherapy prior to surgery; aged 40–80; patients did not suffer from other severe diseases. All patients (29 males, 14 females; mean age, 63 years; age range, 53–79 years) who underwent percutaneous needle biopsies prior to surgery provided informed consent for the biopsy. The subjects were pathologically diagnosed with primary LC according to the AJCC-TNM staging system, 7th edition (Edge et al., 2010). The detailed information of all enrolled patients is presented in Table 1. Normal tissue adjacent to the tumor was collected at a distance of at least 2 cm away from the cancerous tissue. The specimens were rapidly frozen in liquid nitrogen.

**TABLE 1:** Clinicopathological features of the 43 patients with lung cancer in the present study.

**Clinicopathological features**	**Cases (%)**
**Age, years**	
≤60	16 (37.2)
>60	27 (62.8)
**Sex**	
Male	29 (67.4)
Female	14 (32.6)
**Smoking status**	
Yes	35 (81.4)
No	8 (18.6)
**Tumor-node-metastasis stage**	
I	17 (39.5)
II	15 (34.9)
III	11 (25.6)
**Pathological type**	
Adenocarcinoma	26 (60.5)
Squamous carcinoma	17 (39.5)
**Lymph node metastasis**	
Yes	32 (74.4)
No	11 (25.6)
**Differentiation degree**	
High	9 (20.9)
Moderate	21 (48.8)
Low	13 (30.3)

### Cells and Cell Culture

Human lung adenocarcinoma cell lines A549 (Cat no. SCSP-503), NCI-H1975 (Cat no. TCHu193) and NCI-H460 (H460, Cat no. TCHu205), BEAS-2B human bronchial epithelial cells (Cat no. SCSP-5067), and 293T cells (Cat no. SCSP-502) were purchased from the Shanghai Cell Bank, Chinese Academy of Sciences (Shanghai, China). 95-D human lung squamous carcinoma cell line was obtained from the American Type Culture Collection (ATCC). EBC-1 human lung squamous carcinoma cell line was obtained from the Japanese Research Resources Bank (Tokyo, Japan). BEAS-2B cells are normal lung epithelial cells, and the BEAS-2B cell line is regarded as a “control” for LC cells. In follow-up experiments, the present study mainly focused on using the A549 and H1975 cell lines, as the clinicopathological types of LC represented by these two cell lines are more common. A549, H1975, H460, 95D, and EBC-1 cells were cultured in RPMI-1640 medium (Gibco, Thermo Fisher Scientific, Inc., Cat no. 11875093) containing 10% fetal bovine serum (Gibco, Cat no. 16140071) and 1% penicillin-streptomycin (Gibco, Cat no. 15140148). BEAS-2B normal lung epithelial and 293T cells were cultured using DMEM (Gibco, Cat no. 11965092) supplemented with 10% fetal bovine serum and 1% penicillin-streptomycin. All cells were maintained in an incubator at 37°C, in an atmosphere with 5% CO_2_ and saturated humidity.

### Plasmid Construction

The coding region (CDS) of c-Met was amplified and cloned into the multiple cloning sites of plasmid pcDNA3.1 (Promega Corporation). Thereafter, 2 μg of the plasmids were transfected into 293T cells using Lipofectamine^TM^ 3000 transfection reagent (Thermo Fisher Scientific, Inc., #L3000015). The transduction efficiency was examined by RT-qPCR or western blotting 24 h post-transfection.

### Construction of Linc00284-Knockdown LC Cell Lines

The lentivirus was produced using the 3rd generation system. The packaging plasmids (*pMDL*, *p-VSV-G*, and *p-REV*) were purchased from Shanghai GeneChem Co., Ltd. Short hairpin RNA (shRNA) targeting Linc00284 (5′-GGGGTCCTGCTGAGCCAGGA-3′) was designed and synthesized by Sangon Biotechnology (Sangon BioTech). The sequences were then cloned into the polyclonal sites of the plasmid *pLVX-puro* vectors (Clontech, Takara Bio Inc., Cat no. 632159). Lentiviral particles were generated by co-transfection of the 293T cells with the shRNA constructs in *pLVX-puro* vectors and the packaging plasmids using polyethyleneimine (Invitrogen, Thermo Fisher Scientific, Inc.) according to the manufacturer’s protocol.

Specifically, 293T cells were seeded in a six-well plate and then transfected with the indicated plasmids as described below. The transfection mixture (100 μl) contained 0.75 μg of *pMDL*, 0.45 μg of *p-VSV-G*, and 0.3 μg of *p-REV* plasmids at a ratio of 5:3:2, 1.5 μg of shRNA vectors and 6 μl of PEI (10 μM), followed by incubation for 30 min at room temperature. Subsequently, an additional 900 μl of complete medium were added to the mixture (PEI, 1 μM). Thereafter, 1 ml of the mixture was carefully added to the cultured 293T cells. At 12 h post-transfection, the medium was replaced with fresh medium. Viral supernatant was collected after 48 h and concentrated by ultracentrifugation (25,000 × *g* for 2.5 h at 4°C). To further construct the stably transfected cell lines, the supernatant was used for the infection of LC cells by mixing with fresh medium at a 1:1 volume ratio; moreover, polybrene (Shanghai GeneChem Co., Ltd.) was added at a final concentration of 10 μg/ml. The plate was centrifuged at 400 × *g*, 37°C for 30 min. Lentiviral infection was performed at a multiplicity of infection (MOI) of approximately 2. At 72 h following infection, the cancer cells were selected with 2 μg/ml of puromycin (Beyotime Institute of Biotechnology, Cat no. ST551) for 7 consecutive days.

### Cell Proliferation Assay

Following transduction, the cells from each group were seeded in six-well plates at a density of 5 × 10^4^ cells/ml, and 10% CCK-8 reagent was added at the corresponding time points according to the instructions provided by the manufacturer (Beijing Solarbio Science and Technology Co., Ltd., Cat no. CK04-500T), following incubation for 2 h at 37°C. The absorbance at 450 nm was measured using an Enzyme-Labeling instrument (Bio-Rad Laboratories, Inc.).

### Transwell Migration and Invasion Assays

Following digestion with 0.25% trypsin-EDTA (Gibco, Cat no. 25200072) and centrifugation at 140 × *g* for 5 min at room temperature, the cells were resuspended using serum-free medium and diluted to a final concentration of 1 × 10^5^ cells/ml. For transwell migration assays, the cells were seeded into the upper chamber of 24-well Transwell plates (Costar, Corning, Inc.), and 500 μl of complete medium were added to the lower chamber. After 12 h, the cells in the lower chamber were fixed and stained with 0.1% crystal violet (Beyotime Institute of Biotechnology, Cat no. C0121) at 37°C for 15 min. Images were obtained with an inverted microscope (Nikon Corporation). A total of five fields of view were randomly selected to count the number of migrated cells. For transwell invasion assay, 100 μl of Matrigel (Corning, Inc., Cat no. 356234) at a final concentration of 2 mg/ml were added to the upper chamber and incubated at 37°C for 30 min. Subsequently, the LC cells were processed as described above.

### Dual Luciferase Reporter Assay

The wild-type (WT) Linc00284 sequence (WT 00284) and the 3′UTR sequence of WT c-Met (WT c-Met 3′UTR) were cloned into the multiple cloning sites of the pmirGLO dual luciferase reporter vector plasmids (Promega Corporation, Cat no. E1330). Mutant Linc00284 and mutant c-Met 3′UTR plasmids were constructed by mutating Linc00284 and c-Met 3′UTR to prevent binding. After 48 h, the cells were lysed and firefly luciferase activity in each group was measured following the manufacturers’ instructions (Dual-Luciferase^®^ Reporter Assay System, Promega Corporation, Cat no. E1910). Firefly luciferase activity was normalized to *Renilla* luciferase activity. For each group, luciferase activity was averaged from six replicates.

### Bioinformatics Analysis

To identify the downstream targets of Linc00284, DIANA-LncBase Predicted v.2^1^ was used and the potential binding sites between Linc00284 and microRNAs were predicted. In addition, to determine the downstream target of miR-205-3p, TargetScan v7.2^2^ was used in the present study. Next, the microRNA and gene of interest were selected based on the published literature.

### Wound Healing Assay

Lung cancer cells were inoculated into six-well plates at a density of 1 × 10^6^ cells/well. After cell confluence reached 90%, the medium was discarded and cells were starved in serum-free medium for 12 h. Thereafter, a sterile 200 μl pipette tip was used to scratch the LC cell monolayer. Floating cells were removed by rinsing with PBS. The cells were maintained in growth medium containing 2% fetal bovine serum. Wound closure gap was photographed under an optical microscope (Nikon Corporation) and was monitored at different time points (0 and 48 h).

### RT-qPCR Analysis

Total RNA was extracted from the LC cells or tumor tissues using pre-cooled TRIzol^®^ reagent (Life Technologies; Thermo Fisher Scientific, Inc., Cat no. 15596026) according to the instructions provided by the manufacturer. Complementary DNA (cDNA) was synthesized using High-Capacity cDNA Reverse Transcription kit (Applied Biosystems; Thermo Fisher Scientific, Inc., #4368813). RT-qPCR was performed using GoTaq^®^ qPCR Master mix (Promega Corporation, Cat no. A6001) following the manufacturer’s instructions and using ABI 7500 System (Applied Biosystems). Relative gene expression levels were calculated using the 2^–ΔΔCq^ method (Livak and Schmittgen, 2001) and normalized to the housekeeping gene β-actin. U6 served as the internal control for the miRNA expression. All primer sequences used in the present study are presented in Table 2.

**TABLE 2:** Primer sequences for RT-qPCR.

**Gene**	**Primer sequences (5′-3′)**	
	**Forward**	**Reverse**
*LINC00284*	CCAGGGGATAAAACCCGCTT	TAAGCACCAAGTCACGCTGT
*CDK4*	CTGGTGTTTGAGCATGTAGACC	GATCCTTGATCGTTTCGGCTG
*CDK6*	TCTTCATTCACACCGAGTAGTGC	TGAGGTTAGAGCCATCTGGAAA
*Cyclin D1*	CAATGACCCCGCACGATTTC	CATGGAGGGCGGATTGGAA
*Bad*	CCCAGAGTTTGAGCCGAGTG	CCCATCCCTTCGTCGTCCT
*Bcl2*	GGTGGGGTCATGTGTGTGG	CGGTTCAGGTACTCAGTCATCC
*Bax*	CCCGAGAGGTCTTTTTCCGAG	CCAGCCCATGATGGTTCTGAT
*N-Cadherin*	AGCCAACCTTAACTGAGGAGT	GGCAAGTTGATTGGAGGGATG
*E-Cadherin*	ATTTTTCCCTCGACACCCGAT	TCCCAGGCGTAGACCAAGA
*Cytokeratin-19*	ACCAAGTTTGAGACGGAACAG	CCCTCAGCGTACTGATTTCCT
*Vimentin*	GACGCCATCAACACCGAGTT	CTTTGTCGTTGGTTAGCTGGT
*MMP-9*	TGTACCGCTATGGTTACACTCG	GGCAGGGACAGTTGCTTCT
*MMP-2*	GATACCCCTTTGACGGTAAGGA	CCTTCTCCCAAGGTCCATAGC
β*-actin*	AATCTGGCACCACACCTTCTAC	TATCGTGTCGGACCTATCGTTG
*c-Met*	AGCGTCAACAGAGGGACCT	GCAGTGAACCTCCGACTGTATG
*microRNA-205-3P*	GATTTCAGTGGAGTGAAGTTC	GTGGAGTCGGCAATTCAGTT
*U6*	ATTGGAACGATACAGAGAAGATT	GGAACGCTTCACGAATTTG

### Western Blotting

Protein lysates from tissues or cultured cells were prepared using pre-cooled RIPA buffer (KeyGEN BioTECH, Cat no. KGP702) containing 1% phenylmethanesulfonyl fluoride (PMSF) (KeyGEN BioTECH, Cat no. KGP610). Protein concentration was determined using the BCA protein assay kit (KeyGEN BioTECH, Cat no. KGP902). Next, heat-denatured proteins were electrophoresed in 10–12% SDS-PAGE gels and transferred onto methanol-activated PVDF membranes (Millipore, Cat no. IPVH00010) by wet transfer electrophoresis. The membranes were then blocked with 5% BSA for 2 h at room temperature, followed by overnight incubation at 4°C with the corresponding primary antibody. After washing with TBST, the membranes were incubated with diluted secondary antibody at room temperature for 2 h. After further TBST washing, HRP-conjugated secondary antibody was added for 2 h at room temperature. Immunoblots were visualized using an enhanced chemiluminescence (ECL) detection system (Millipore, Thermo Fisher Scientific, Inc., Cat no. CPS350) as described by the manufacturer. β-actin was used as an internal control. Data were analyzed using ImageJ software (version 1.52v, National Institutes of Health).

Antibodies used are as follows: Anti-Bax (Abcam, ab182734, dilution, 1:1,000), anti-Bad (Abcam, ab32445, dilution, 1:2,000), anti-Bcl-2 (Abcam, ab182858, dilution, 1:2,000), anti-Cyclin D1 (Abcam, ab40754, dilution, 1:2,000), anti-CDK6 (Abcam, ab241554, dilution, 1:2,000), anti-CDK4 (Abcam, ab108357, dilution, 1:2,000), anti-cytokeratin 19 (Abcam, ab76539, dilution, 1:2,000), anti-E cadherin (Abcam, ab1416, dilution, 1:1,000), anti-Vimentin (Abcam, ab92547, dilution, 1:2,000), anti-matrix metalloproteinase-9 (MMP-9) (Abcam, ab137867, dilution, 1:1,000), anti-MMP2 (Abcam, ab181286, dilution, 1:1,000), anti-N cadherin (Abcam, ab245117, dilution, 1:1,000), anti-c-Met (Abcam, ab51067, dilution, 1:1,000), β-actin (Abcam, ab115777, dilution, 1:1,000), and goat anti-rabbit IgG H&L (HRP) (Abcam, ab205718, dilution, 1:10,000).

### Analysis of Transfection Efficiency

MiR-205-3p mimics and inhibitor were synthesized by Sangon Biotechnology. RNAiMAX transfection reagent was purchased from Sangon Biotechnology. All sequences are listed as follows: miR-205-3p mimics, 5′-GATTTCAGTGGAG TGAAGTT-3′; mimics negative control, 5′-AAGAGCACCAAC TCGAGTTG-3′; miR-205-3p inhibitor, 5′-AACTTCA CTCCACTGAAATC-3′; inhibitor negative control, 5′-GTTGGTGCTCTTCATCTTGTTG-3′. Briefly, 1 μg/ml of nucleic acid was used to transfect LC cells and incubated with RNAiMAX transfection reagent at 37°C for 6 h. Subsequent experiments were performed 48 h following transfection. Thereafter, the mRNA/protein expression of miR-205-3p or c-Met in A549 or H1975 cells transfected with miR-205-3p mimics, inhibitor or OE-c-Met was evaluated by RT-qPCR and/or western blot analysis (Supplementary Figure 1).

### Flow Cytometry Analysis

Cell Cycle and Apoptosis Analysis kit was purchased from Beyotime Institute of Biotechnology (Cat no. C1052). For apoptosis analysis, the cells were stained with Annexin V-FITC and propidium iodide (PI). For cell cycle analysis, the cells were stained with PI. The stained cells were then assessed by flow cytometry following the manufacturers’ instructions on a BD FACSAria II flow cytometer (BD Biosciences). Data were analyzed using FlowJo software (FlowJo LLC, version 10.6.0).

### Tumor Formation in Nude Mice Following Subcutaneous Injection

All animal experiments were performed with the approval of the Institutional Animal Care and Use Committee of The First Affiliated Hospital of Fujian Medical University (approval no. FMU-ACUC17-141). Male athymic BALB/c nude mice (6 weeks, approximately 20 g in body weight) were purchased from the Model Animal Research Center of Nanjing University (Nanjing, China). A total of 24 mice were randomly divided into four groups (*n* = 6 mice per group). Following 1 week of acclimatization, the mice received 1 × 10^6^ tumor cells (H1975 or A549 cells) subcutaneously in each flank. Tumor diameters were monitored weekly following implantation using a caliper. The mice were sacrificed after 5 or 6 weeks. Tumors were resected and tumor mass was determined. Tumor volumes were calculated using the following formula: (length × width^2^)/2.

### Immunohistochemistry

Tissues were fixed, dehydrated, paraffin-embedded, sectioned at 6 μm thickness, dewaxed, rehydrated, subjected to antigen retrieval and incubated overnight with primary antibodies: anti-c-Met (Abcam, ab51067, 1:100 dilution); anti Ki-67 (Abcam, ab16667, 1:200 dilution) at 4°C. After washing with PBS, the sections were incubated with goat anti-rabbit IgG H&L (HRP) antibody (Abcam, ab205718, 1:20,000 dilution) for 2 h at room temperature. After further PBS washing, the sections were subjected to 3, 3-diaminobenzidine (DAB, Beyotime Institute of Biotechnology, Cat no. P0203) staining for 10 min at room temperature followed by hematoxylin counterstaining for 2 min at room temperature. The sections were examined by an optical microscope (Nikon Corporation).

### Statistical Analysis

Data analysis was performed using SPSS 21.0 statistical software (SPSS, Inc.). Results are expressed as the means ± standard deviation (SD) of triplicate independent experiments. Comparisons between two groups was performed using an unpaired *t*-test. The expression of Linc00284, miR-205-3p, and c-Met in 43 LC tissues and paired adjacent non-cancerous tissues was analyzed using paired *t*-test. Descriptive statistics were used to analyze the expression of Linc00284 in the cytoplasm and in the nucleus of LC cells. Multiple comparisons were analyzed using one-way analysis of variance (ANOVA) followed by Tukey’s test. Correlation analysis was performed using Pearson’s correlation analysis. The analysis of overall survival was performed using Kaplan–Meier curves (log-rank test). A *P*-value < 0.05 was considered to indicate a statistically significant difference.

## Results

### Upregulation of Linc00284 Is Associated With a Poor Prognosis in Patients With Lung Cancer

**FIGURE 1:**
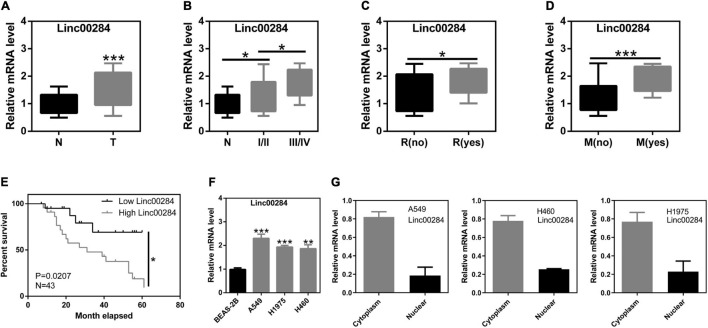
Linc00284 expression is significantly upregulated in lung cancer tissues and is associated with tumor progression. **(A)** Linc00284 expression in 43 lung cancer tissues and patient-matched normal tissues. **(B–E)** Linc00284 expression was associated with **(B)** TNM stage, **(C)** recurrence, **(D)** metastasis, and **(E)** overall survival of patients with lung cancer. **(F)** Linc00284 expression was significantly upregulated in A549, H1975, and H460 lung cancer cells. **(G)** Linc00284 was predominantly observed in the cytoplasm of lung cancer cells. Data are expressed as the means ± standard deviation (SD) for independent triplicate experiments. The expression of Linc00284 in 43 lung cancer tissues and paired adjacent non-cancerous tissues was analyzed using paired *t*-test. Multiple comparisons were analyzed using one-way analysis of variance (ANOVA) followed by Tukey’s test. Kaplan–Meier analysis was used for survival analysis. Descriptive statistics were used to analyze the expression of Linc00284 in the cytoplasm and in the nucleus of LC cells. *P < 0.05, **P < 0.01, and ***P < 0.001, compared with the matched control group. N, adjacent normal tissues; T, tumor tissues; R, recurrence; M, metastasis.

We first determined the expression of Linc00284 in 43 human LC and adjacent normal tissues. Following the extraction of total RNA and performing RT-qPCR, it was found that Linc00284 expression was significantly upregulated in LC tissues compared with adjacent normal samples (Figure 1A). Moreover, Linc00284 expression was higher in Stage III-IV LC tissues than in Stage I-II LC tissues (Figure 1B). Besides, Linc00284 was highly expressed in tumor tissues from lung cancer patients with metastasis/recurrence compared to those patients without metastasis/recurrence (Figures 1C,D). The median Linc00284 expression in the LC tissues was used to distinguish between a high and low Linc00284 expression. Kaplan–Meier analysis revealed that LC patients with high Linc00284 expression had a shorter overall survival than those with low Linc00284 expression (Figure 1E). In *in vitro* experiments, the comparison with BESA-2B human normal lung epithelial cells revealed that Linc00284 expression was markedly increased in the LC cells (A549, H1975, and H460) (Figure 1F). And Linc00284 was mainly localized in the cytoplasm of LC cells (Figure 1G).

### Linc00284 Knockdown Contributes to the Inhibition of LC Cell Proliferation

**FIGURE 2:**
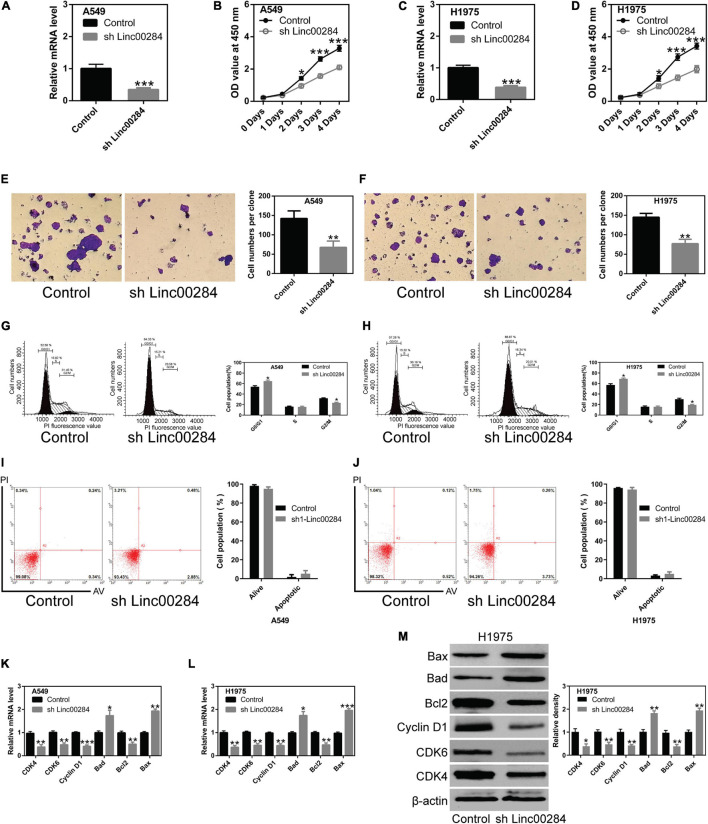
Linc00284 silencing inhibits lung cancer cell proliferation. **(A)** Knockdown efficiency was measured by RT-qPCR in A549 human lung cancer cells. **(B)** The proliferative capacity of A549 cells with Linc00284 silencing was determined by CCK-8 assay. **(C)** Knockdown efficiency was measured by RT-qPCR in H1975 human lung cancer cells. **(D)** Proliferation of Linc00284-silenced H1975 cells was measured by CCK-8 assay. **(E,F)** Representative images and statistical analysis results of the colony-forming ability of Linc00284-silenced **(E)** A549 and **(F)** H1975 cells, as determined by colony formation assay. Magnification, ×10. **(G,H)** Representative images and quantification of cell cycle distribution of Linc00284-silenced **(G)** A549 and **(H)** H1975 cells, as determined by flow cytometry. **(I,J)** Apoptosis of **(I)** A549 and **(J)** H1975 cells in which Linc00284 was knockdown, measured using flow cytometry. **(K,L)** mRNA levels of apoptosis- or cell cycle-related genes in Linc00284-silenced **(K)** A549 and **(L)** H1975 cells, as evaluated by RT-qPCR. **(M)** Apoptosis- or cell cycle-related protein expression in H1975 cells in which Linc00284 was knocked down, determined by western blotting. Data were analyzed using unpaired *t*-test. Error bar represents standard deviation (SD) for triplicate independent experiments. *P < 0.05, **P < 0.01, and ***P < 0.001, compared with matched control cells.

To understand the biological function of Linc00284 in LC cells, we constructed lentivirus-mediated Linc00284-silenced LC cell lines. The efficiency of Linc00284 knockdown was evaluated by RT-qPCR analysis (Figures 2A,C). The effect of Linc00284 silencing on LC cell proliferation was also assessed. Linc00284 silencing significantly inhibited LC cell proliferation (Figures 2B,D and Supplementary Figure 2) and colony formation (Figures 2E,F). Flow cytometric analysis further revealed that Linc00284 silencing caused G0/G1 phase cell cycle arrest (Figures 2G,H), but had no marked effect on cell apoptosis (Figures 2I,J). Moreover, Linc00284 knockdown induced down-regulation of cell cycle-related genes (*CDK4*, *CDK6*, and *cyclin D1*) and pro-survival gene *Bcl-2* in the A549 and H1975 cells, while Linc00284 knockdown upregulated the expression of apoptosis-related genes *Bad* and *Bax* at the transcriptional and translational levels (Figures 2K–M).

### Linc00284 Silencing Suppresses the Migration and Invasion of LC Cells

**FIGURE 3:**
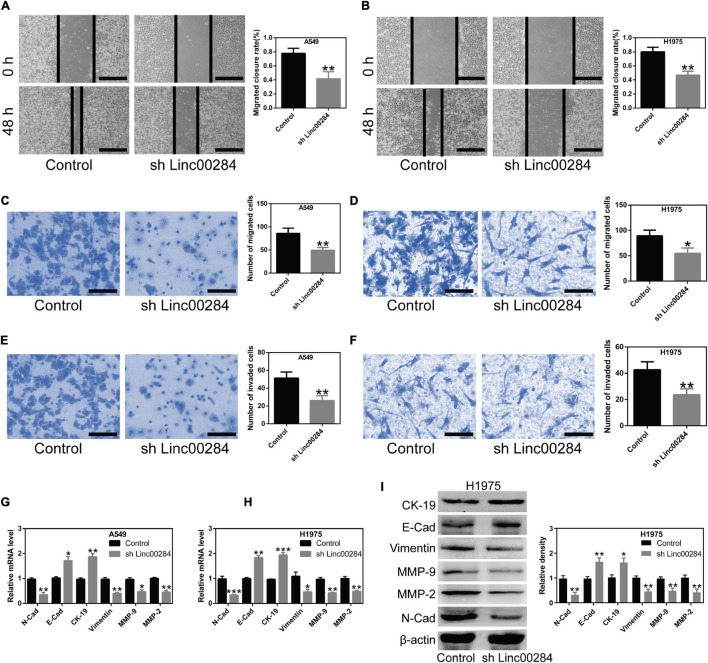
Linc00284 silencing suppresses lung cancer cell migration and invasion. **(A,B)** Representative images and statistical results of migratory ability of Linc00284-silenced **(A)** A549 and **(B)** H1975 cells as determined by wound healing assays. Magnification, ×4. **(C,D)** Transwell migration assay was performed to examine migratory ability of Linc00284-silenced **(C)** A549 and **(D)** H1975 cells. Magnification, ×40. **(E,F)** Transwell invasion assays were performed to detect invasive capabilities of Linc00284-silenced **(E)** A549 and **(F)** H1975 cells. Magnification, ×40. **(G,H)** mRNA levels of EMT-related genes in **(G)** A549 and **(H)** H1975 cells. **(I)** Protein expression of EMT-related genes in H1975 cells. Data were analyzed using unpaired *t*-test and are expressed as the means ± SD for triplicate independent experiments. *P < 0.05, **P < 0.01, and ***P < 0.001, compared with the matched control cells. EMT, epithelial-mesenchymal transition.

The present study then examined the effects of Linc00284 silencing on cell migratory and invasive abilities using wound healing and transwell assays. Results revealed that Linc00284 silencing significantly suppressed the migratory (Figures 3A–D) and invasive (Figures 3E,F and Supplementary Figure 2) capabilities of LC cells. Additionally, RT-qPCR and western blotting analyses confirmed that Linc00284 knockdown contributed to the significant upregulation of genes associated with the epithelial phenotype (*E-Cadherin* and *cytokeratin 19*) and to the downregulation of genes related to the mesenchymal phenotype (*N-Cadherin*, *Vimentin*, *MMP-9*, and *MMP-2*) (Figures 3G–I). These findings indicate that Linc00284 plays a role in promoting the migration and invasion of LC cells.

### Linc00284 Directly Binds to miR-205-3p

**FIGURE 4:**
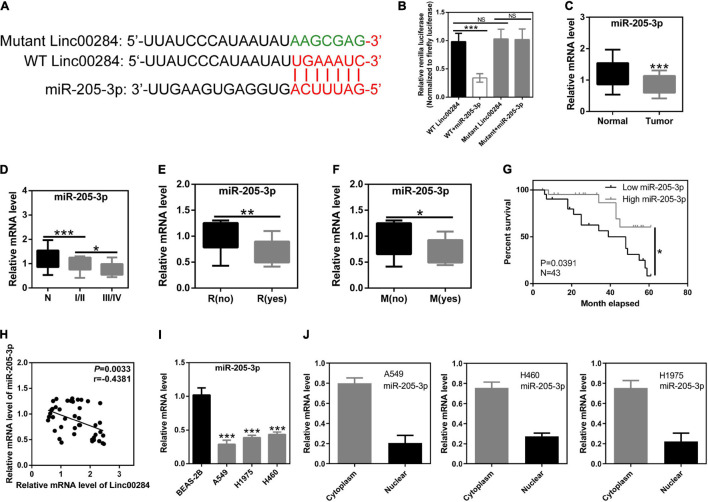
Linc00284 directly targets miR-205-3p. **(A)** Schematic representation of direct binding sites between Linc00284 and miR-205-3p. Red indicates the predicted binding sites between Linc00284 and miR-205-3p. Green indicates the mutant sequence of Linc00284. **(B)** Dual-luciferase reporter assay confirmed direct interaction between miR-205-3p and Linc00284. **(C)** RT-qPCR revealed the significant downregulation of miR-205-3p in lung cancer tissues. **(D–G)** miR-205-3p expression was associated with **(D)** TNM stage, **(E)** recurrence, **(F)** metastasis, and **(G)** overall survival of patients with lung cancer. **(H)** Pearson’s correlation analysis indicated that miR-205-3p expression was negatively correlated with the Linc00284 level in tumor tissues. **(I)** miR-205-3p expression was significantly upregulated in A549, H1975, and H460 lung cancer cell lines. **(J)** miR-205-3p was mainly located in the cytoplasm of lung cancer cells. Data are expressed as the means ± SD for triplicate independent experiments. The expression of miR-205-3p in 43 lung cancer tissues and paired adjacent non-cancerous tissues was analyzed using paired *t*-test. Multiple comparisons were analyzed using one-way ANOVA followed by Tukey’s test. Kaplan–Meier analysis was used for survival analysis. Correlation analysis was assessed using Pearson’s correlation analysis. Descriptive statistics were used to analyze the expression of miR-205-3p in the cytoplasm and in the nucleus of LC cells. *P < 0.05, **P < 0.01, and ***P < 0.001, compared with the matched control group. NS, not significant. N, adjacent normal tissues; T, tumor tissues; R, recurrence; M, metastasis.

To identify the downstream targets of Linc00284, LncBase Predicted V2.0 software was used and potential binding sites were identified between Linc00284 and various miRNAs, including miR-205-3p (Figure 4A). Importantly, a previous study demonstrated that miR-205-3p functions as tumor suppressor in ovarian cancer (Qiao et al., 2020) and low tumor miR-205-3p expression levels was associated with poor prognosis in patients with gastric cancer (Zhang et al., 2020). Furthermore, the present study demonstrated that Linc00284 was significantly upregulated in LC tissues compared with adjacent normal samples. In addition, compared with BESA-2B normal lung epithelial cells, Linc00284 expression was markedly increased in LC cells (A549, H1975, and H460), as described above. Therefore, miR-205-3p was selected as the target of Linc00284. The interaction between Linc00284 and miR-205-3p was confirmed using a dual luciferase reporter assay. Transfection with miR-205-3p mimics significantly reduced the luciferase activity in 293T cells transfected with WT Linc00284 plasmids, but had no effect on luciferase activity in 293T cells transfected with mutant Linc00284 plasmids (Figure 4B). Additionally, RT-qPCR analysis showed that miR-205-3p expression was significantly decreased in LC tissues (Figure 4C) and was associated with TNM stage, metastasis, and tumor recurrence (Figures 4D–F). LC patients with high miR-205-3p expression had a favorable prognosis for survival (Figure 4G). Moreover, the miR-205-3p level was negatively correlated with Linc00284 expression (Figure 4H). miR-205-3p expression was also found to be downregulated in LC cells (Figure 4I) and miR-205-3p was mainly localized in the cytoplasm (Figure 4J).

### c-Met Is the Downstream Target of miR-205-3p

**FIGURE 5:**
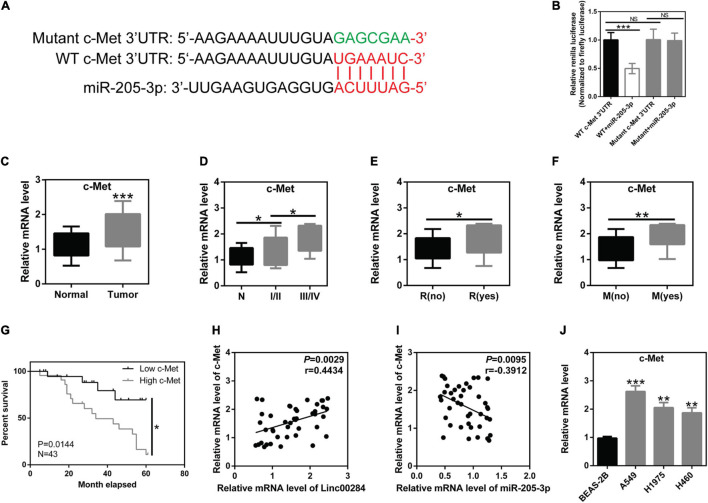
c-Met is the direct target of miR-205-3p. **(A)** Schematic diagram of the binding sites between miR-205-3p and the 3′UTR of c-Met mRNA. Red indicates the predicted binding sites between c-Met and miR-205-3p. Green indicates the mutant sequence of c-Met. **(B)** A direct interaction between miR-205-3p and the 3′UTR of c-Met mRNA. **(C)** Significant upregulation of c-Met in 43 human lung cancer tissues and patient-matched normal tissues. **(D–G)** c-Met expression was associated with **(D)** TNM stage, **(E)** recurrence, **(F)** metastasis, and **(G)** overall survival of patients with lung cancer. **(H)** Pearson’s correlation analysis revealed that c-Met expression was positively correlated with the Linc00284 level in human lung cancer tissues. **(I)** c-Met expression was negatively correlated with the miR-205-3p level in human lung cancer tissues. **(J)** mRNA levels of c-Met were significantly upregulated in A549, H1975, and H460 lung cancer cell lines. Data are expressed as the means ± SD of triplicate independent experiments. The expression of c-Met in 43 lung cancer tissues and paired adjacent non-cancerous tissues was analyzed using paired *t*-test. Multiple comparisons were analyzed by one-way ANOVA followed by Tukey’s test. Kaplan–Meier analysis was used for survival analysis. Correlation analysis was assessed using Pearson’s correlation analysis. *P < 0.05, **P < 0.01, and ***P < 0.001, compared with the matched control group. NS, not significant. N, adjacent normal tissues; T, tumor tissues; R, recurrence; M, metastasis.

In this study, further analysis revealed that the 3′UTR of c-Met mRNA may be a direct target of miR-205-3p (Figure 5A). c-Met, a well-known oncogene, represents an attractive therapeutic target (Ai et al., 2018). Dual luciferase reporter experiments confirmed the direct interaction between the 3′UTR of c-Met mRNA and miR-205-3p (Figure 5B). In clinical samples, c-Met expression was markedly upregulated in LC tissues compared to adjacent normal tissues (Figure 5C). Moreover, c-Met expression was associated with TNM stage, distant metastasis, and tumor recurrence (Figures 5D–F). LC patients with high c-Met expression had a worse prognosis (Figure 5G). c-Met expression was also found to positively correlate with the Linc00284 level (Figure 5H), whereas c-Met expression was significantly negatively correlated with the miR-205-3p level (Figure 5I). *In vitro*, c-Met expression was also increased in LC cells (A549, H1975, and H460) (Figure 5J). Collectively, these data indicate that the Linc00284/miR-205-3p/c-Met axis is implicated in LC progression.

### Linc00284/miR-205-3p/c-Met Axis Modulates Malignant Phenotypes of LC Cells

**FIGURE 6:**
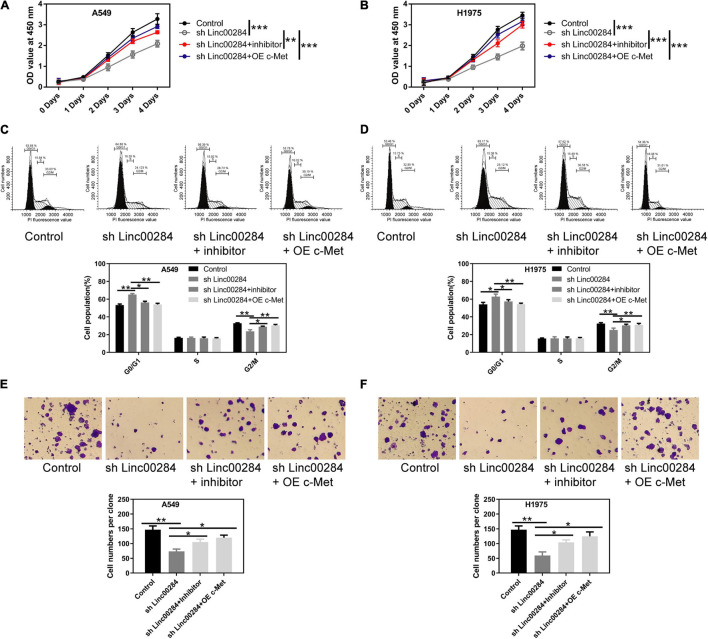
Linc00284/miR-205-3p/c-Met axis regulates lung cancer cell proliferation. **(A,B)** Cell viability of **(A)** A549 and **(B)** H1975 cells in which Linc00284 was knocked down with miR-205-3p silencing or c-Met overexpression as assessed by CCK-8 assay. **(C,D)** Representative images and statistical results of cell cycle distribution in **(C)** A549 and **(D)** H1975 cells in which Linc00284 was knocked down with miR-205-3p silencing or c-Met overexpression. **(E,F)** Representative images and quantification of the colony-forming abilities of **(E)** A549 and **(F)** H1975 cells in which Linc00284 was knocked down with miR-205-3p silencing or c-Met overexpression. Magnification, ×10. Data are expressed as the means ± SD of triplicate independent experiments. Multiple comparisons were analyzed using one-way ANOVA followed by Tukey’s test. *P < 0.05, **P < 0.01, and ***P < 0.001, compared with the matched control cells.

**FIGURE 7:**
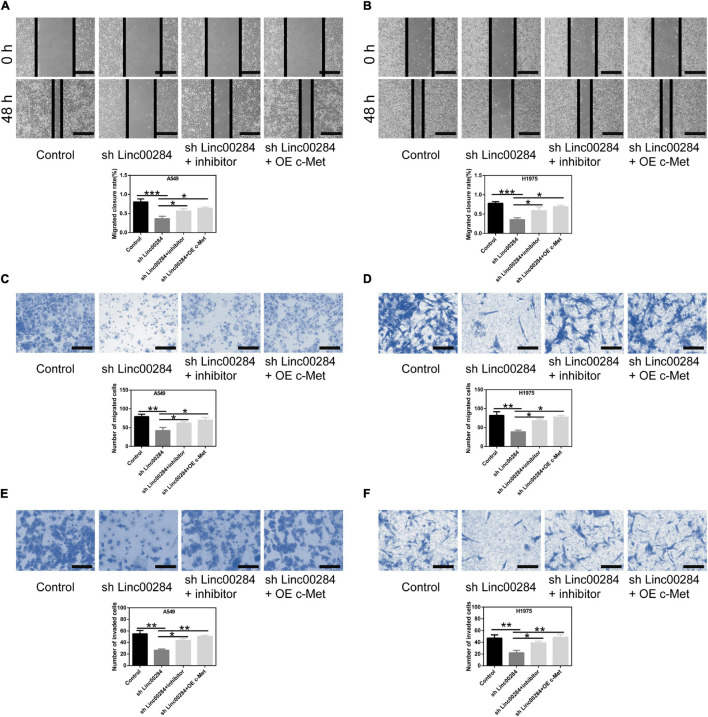
Linc00284/miR-205-3p/c-Met regulatory axis modulates lung cancer cell migration and invasion. **(A,B)** Wound healing assays were used to test the migratory abilities of **(A)** A549 and **(B)** H1975 cells in which 2Linc00284 was knocked down with miR-205-3p silencing or c-Met overexpression. Magnification, ×4. **(C,D)** Transwell migration assays were performed to evaluate the migratory capabilities of **(C)** A549 and **(D)** H1975 cells in which Linc00284 was knocked down with miR-205-3p silencing or c-Met overexpression. Magnification, ×20. **(E,F)** Transwell invasion assays were conducted to detect the invasive capabilities of **(E)** A549 and **(F)** H1975 cells in which Linc00284 was knocked down with miR-205-3p silencing or c-Met overexpression. Magnification, ×40. 0Data are expressed as the means ± SD of triplicate independent experiments. Multiple comparisons were analyzed using one-way ANOVA followed by Tukey’s test. *P < 0.05, **P < 0.01, and ***P < 0.001, compared with the matched control cells.

To examine the role of the Linc00284/miR-205-3p/c-Met axis in LC cells, cell proliferation, colony formation, and transwell assays were performed. We found that Linc00284 silencing attenuated the proliferative (Figures 6A–D), colony-forming (Figures 6E,F), migratory (Figures 7A–D), and invasive (Figures 7E,F) abilities of LC cells. However, the introduction of miR-205-3p inhibitor or c-Met overexpression reversed the effects of Linc00284 knockdown on A549 and H1975 cells. These data thus suggest that the Linc00284/miR-205-3p/c-Met axis promotes the proliferation, migration and invasion of LC cells.

### Linc00284 Knockdown Restrains LC Cell Growth *in vivo*

**FIGURE 8:**
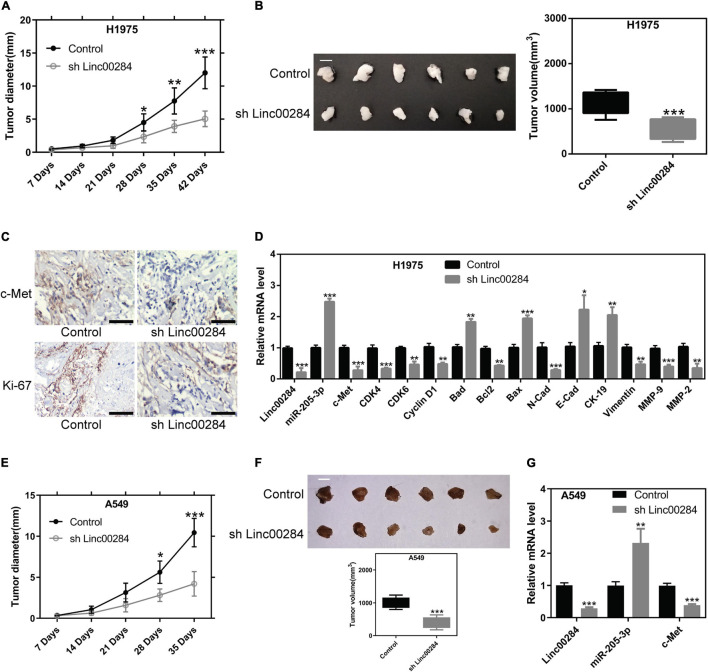
Linc00284 silencing suppresses tumor growth *in vivo*. **(A)** Statistical analysis of the tumor diameter at different time points following implantation (*n* = 6). **(B)** Representative images and quantification of tumor volume at 42 days following implantation. Scale bar, 1 cm. **(C)** Immunohistochemistry was performed to detect c-Met and Ki-67 expression levels in tumor tissues. Magnification, ×40. **(D)** mRNA levels of cell cycle- and EMT-related genes in tumor tissues from H1975 lung cancer cell-bearing mice. **(E)** Tumor diameter was monitored in H1975-bearing nude mice (*n* = 6). Scale bar, 1 cm. **(F)** Tumor volume 35 days following implantation. **(G)** Linc00284, mi5R-205-3, and c-Met expression in tumor tissues. Data were analyzed using unpaired *t*-test and are expressed as the means ± SD. *P < 0.05, **P < 0.01, and ***P < 0.001, compared with the control group. EMT, epithelial-mesenchymal transition.

To further validate the effects of Linc00284 on cell proliferation *in vivo*, mouse xenograft experiments were performed using H1975 and A549 cells. H1975 or A549 cells in which Linc00284 was silenced and matched control cells were subcutaneously injected into immunodeficient mice, and the tumor diameter was measured weekly. Results demonstrated that Linc00284 silencing significantly inhibited the proliferative capacity of the H1975 (Figures 8A,B) and A549 (Figures 8E–G) cells *in vivo*. Immunohistochemistry revealed that Ki-67 and c-Met expression levels were lower in tumor tissues derived from cells in which Linc00284 was silenced compared with the control group (Figure 8C). RT-qPCR analysis also revealed that the expression levels of cell cycle-related and epithelial-mesenchymal transition (EMT)-related genes were consistent with the results obtained *in vitro* (Figure 8D). Overall, these data confirm that Linc00284 plays a promoting role in LC progression.

## Discussion

In the present study, our findings indicated that Linc00284 expression was significantly upregulated in LC tissues compared with patient-matched normal tissues. LC patients with high Linc00284 expression had an unfavorable prognosis. Consistent with the findings of previous studies (Xing et al., 2018; Zhao et al., 2018; Wang et al., 2020b), Linc00284 was predominantly localized in the cytoplasm, suggesting that Linc00284 may act as an endogenous miRNA sponge. Further data confirmed that Linc00284 directly targeted miR-205-3p and downregulated the miR-205-3p levels, causing the upregulation of the c-Met proto-oncogene. The Linc00284/miR-205-3p/c-Met axis promoted the proliferation, migration, and invasion of LC cells *in vitro*. Linc00284 knockdown inhibited tumor growth *in vivo*. Taken together, these results demonstrate that targeting Linc00284 may represent a novel strategy for the treatment of patients with LC.

Linc00284 derives from NRAD1. Consistent with the results obtained for other types of cancer (Xing et al., 2018; Zhao et al., 2018; Ruan and Zhao, 2019; Vidovic et al., 2020; Wang et al., 2020a, b), an exceptionally high Linc00284 expression was also observed in LC, suggesting that Linc00284 may be an important tumor-promoting factor. Moreover, Linc00284 silencing by RNAi suppressed cancer cell proliferation *in vitro* and *in vivo*. In 2017, the United States Food and Drug Administration (FDA) approved antisense oligonucleotides for the treatment of human neurodegenerative disorders (Schoch and Miller, 2017). Targeting Linc00284 appears promising as an effective therapeutic strategy for several types of cancer. Mechanistically, previous research has reported that Linc00284 expression is regulated by aldehyde dehydrogenase 1A3 (ALDH1A3) and its product, retinoic acid (Vidovic et al., 2020). ALDH1A3 is the predominant aldehyde dehydrogenase (ALDH) isozyme responsible for ALDH activity and tumorigenicity in non-small cell LC (Shao et al., 2014). Hence, it was hypothesized that ALDH1A3-mediated Linc00284 is involved in promoting LC progression. Further studies are required to clarify these possibilities.

Based on the competitive endogenous RNA (ceRNA) hypothesis, lncRNAs act as miRNA sponges, which competitively bind to miRNAs and abolish their function (Karreth and Pandolfi, 2013). Previous research has suggested two specific downregulated miRNAs, hsa-miR-195-5p and hsa-miR-497-5p, predicted to interact with LINC00284 in human serous ovarian carcinoma (Wang et al., 2020b). In the present study, LINC00284 was found to mainly function in the cytoplasm, and our data confirmed the interaction between Linc00284 and miR-205-3p. miR-205-3p has been reported to play a tumor-suppressive role in ovarian cancer (Qiao et al., 2020). Additionally, miR-205-3p has been shown to be significantly downregulated in gastric cancer tissues (Zhang et al., 2020). The findings of the present study were consistent with those of previous reports (Qiao et al., 2020), demonstrating a decreased miR-205-3p expression in cancer tissues. Further studies investigating the LINC00284-associated ceRNA network may provide useful information to understand the molecular mechanisms contributing to LC development and progression.

MicroRNAs (miRNAs) are non-coding endogenous RNAs that posttranscriptionally regulate gene expression by binding to the 3′UTRs of mRNAs, leading to cytoplasmic mRNA degradation (Bartel, 2009). In a previous study, miR-205-3p was shown to promote the EMT process by targeting zinc finger electron box binding homologous box 1 (ZEB1) and 2 (ZEB2) (Zhang et al., 2020). Besides, Qiao et al. (2020) identified MAPK10 (JNK3) as a direct target of miR-205-3p, and the upregulation of MAPK10 expression facilitates ovarian cancer cell growth and migration. Here, our data confirmed that c-Met, a ligand of hepatocyte growth factor, was the direct target of miR-205-3p. Linc00284 upregulates c-Met expression by adsorbing miR-205-3p. A large body of data have indicated that c-Met is an oncogene that drives cancer progression (Bouattour et al., 2018; Miranda et al., 2018; Zhang et al., 2018). In rescue experiments, c-Met overexpression reversed the phenotypes caused by Linc00284 knockdown in LC cells, suggesting that Linc00284 exerts its biological function by upregulating c-Met expression. However, whether Linc00284 modulates multiple gene expression by sponging miR-205-3p remains to be further elucidated. In addition, it is certainly required to determine the role of the Linc00284/miR-205-3p/c-Met regulatory axis in LC progression via *in vivo* experiments.

## Conclusion

The present findings reveal that Linc00284 is significantly upregulated in LC tissues and promotes oncogene c-Met expression by directly binding to miR-205-3p. Linc00284/miR- 205-3p/c-Met axis plays cancer-promoting role during LC progression.

## Data Availability Statement

The datasets presented in this study can be found in online repositories. The names of the repository/repositories and accession number(s) can be found in the article/Supplementary Material.

## Ethics Statement

The studies involving human participants were reviewed and approved by the Ethics Committee of The First Affiliated Hospital of Fujian Medical University. The patients/participants provided their written informed consent to participate in this study. The animal study was reviewed and approved by the Institutional Animal Care and Use Committee of The First Affiliated Hospital of Fujian Medical University.

## Author Contributions

FD and RX conceived and designed the study. WS, WG, FL, HL, and RX conducted the experiments and analyzed the data. WS and RX wrote the article. All authors read, edited, and approved the final version of the manuscript.

## Conflict of Interest

The authors declare that the research was conducted in the absence of any commercial or financial relationships that could be construed as a potential conflict of interest.

## Publisher’s Note

All claims expressed in this article are solely those of the authors and do not necessarily represent those of their affiliated organizations, or those of the publisher, the editors and the reviewers. Any product that may be evaluated in this article, or claim that may be made by its manufacturer, is not guaranteed or endorsed by the publisher.

## Footnotes

1
http://carolina.imis.athena-innovation.gr/diana_tools/web/index.php?r=lncbasev2/index-predicted

2
http://www.targetscan.org/vert_72/

## Supplementary Material

Supplementary Figure 1 mRNA/protein expression of miR-205-3p or c-Met in A549 and H1975 cells. **(A)** miR-205-3p level in A549 and H1975 cells transfected with miR-205-3p mimics/inhibitor or the matched control sequences was measured by RT-qPCR. **(B)** mRNA and protein expression of c-Met in A549 and H1975 cells transfected with OE-c-Met or the control vector was detected by RT-qPCR and western blot analysis. Data are expressed as the means ± SD of triplicate independent experiments. Two-group comparisons were analyzed using unpaired *t*-test. ^∗∗∗^*P* < 0.001, compared with the matched control cells.

Supplementary Figure 2 Effects of Linc00284 knockdown on proliferation, migration, and invasiveness of human lung squamous carcinoma cells. **(A–C)** Linc00284 knockdown suppressed the **(A)** proliferation, **(B)** migration, and **(C)** invasion of 95-D human lung squamous carcinoma cells. **(D–F)** Linc00284 knockdown inhibited the **(D)** proliferation, **(E)** migration, and **(F)** invasion of EBC-1 human lung squamous carcinoma cells. Data were analyzed using unpaired *t*-test. Error bar represents standard deviation (SD) for triplicate independent experiments. ^∗∗^*P* < 0.01 and ^∗∗∗^*P* < 0.001, compared with matched control cells.
