# Supplementary figures and images for: Upregulation of Linc00284 Promotes Lung Cancer Progression by Regulating the miR-205-3p/c-Met Axis
Source: Front Genet. 2021 Sep 20;12:694571. doi: 10.3389/fgene.2021.694571 (PMC8488201; doi:10.3389/fgene.2021.694571)

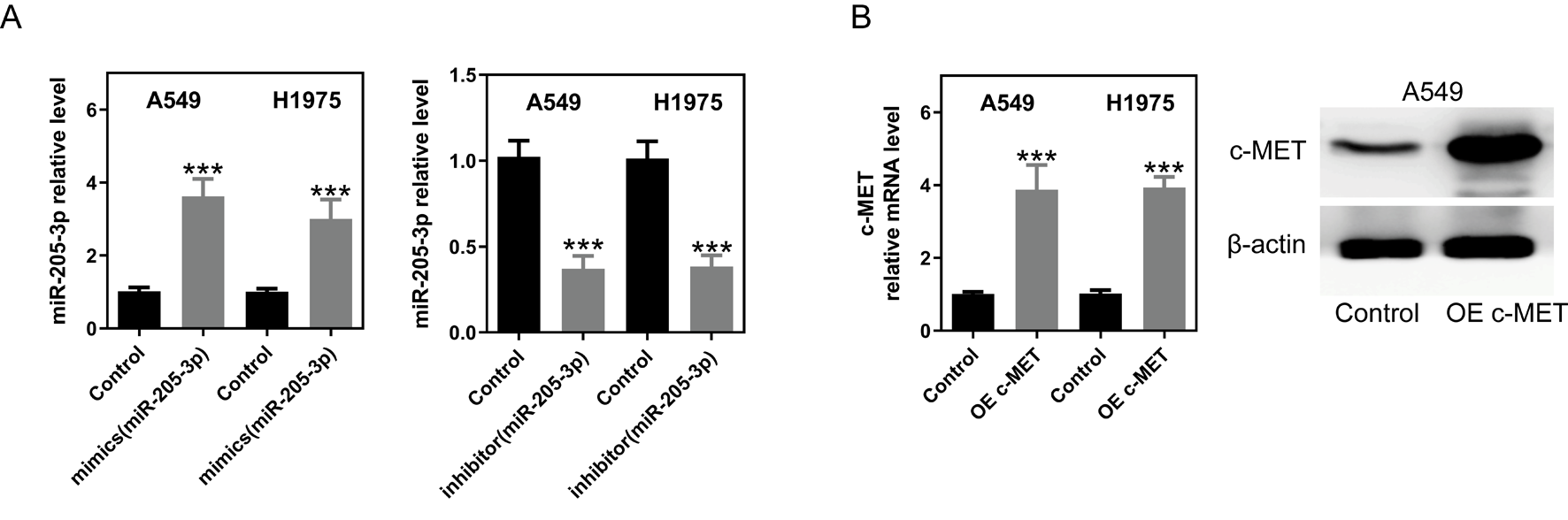

Supplement: Supplementary Figure 1 — mRNA/protein expression of miR-205-3p or c-Met in A549 and H1975 cells. (A) miR-205-3p level in A549 and H1975 cells transfected with miR-205-3p mimics/inhibitor or the matched control sequences was measured by RT-qPCR. (B) mRNA and protein expression of c-Met in A549 and H1975 cells transfected with OE-c-Met or the control vector was detected by RT-qPCR and western blot analysis. Data are expressed as the means ± SD of triplicate independent experiments. Two-group comparisons were analyzed using unpaired t-test. ∗∗∗P < 0.001, compared with the matched control cells. [file Image_1.TIF]

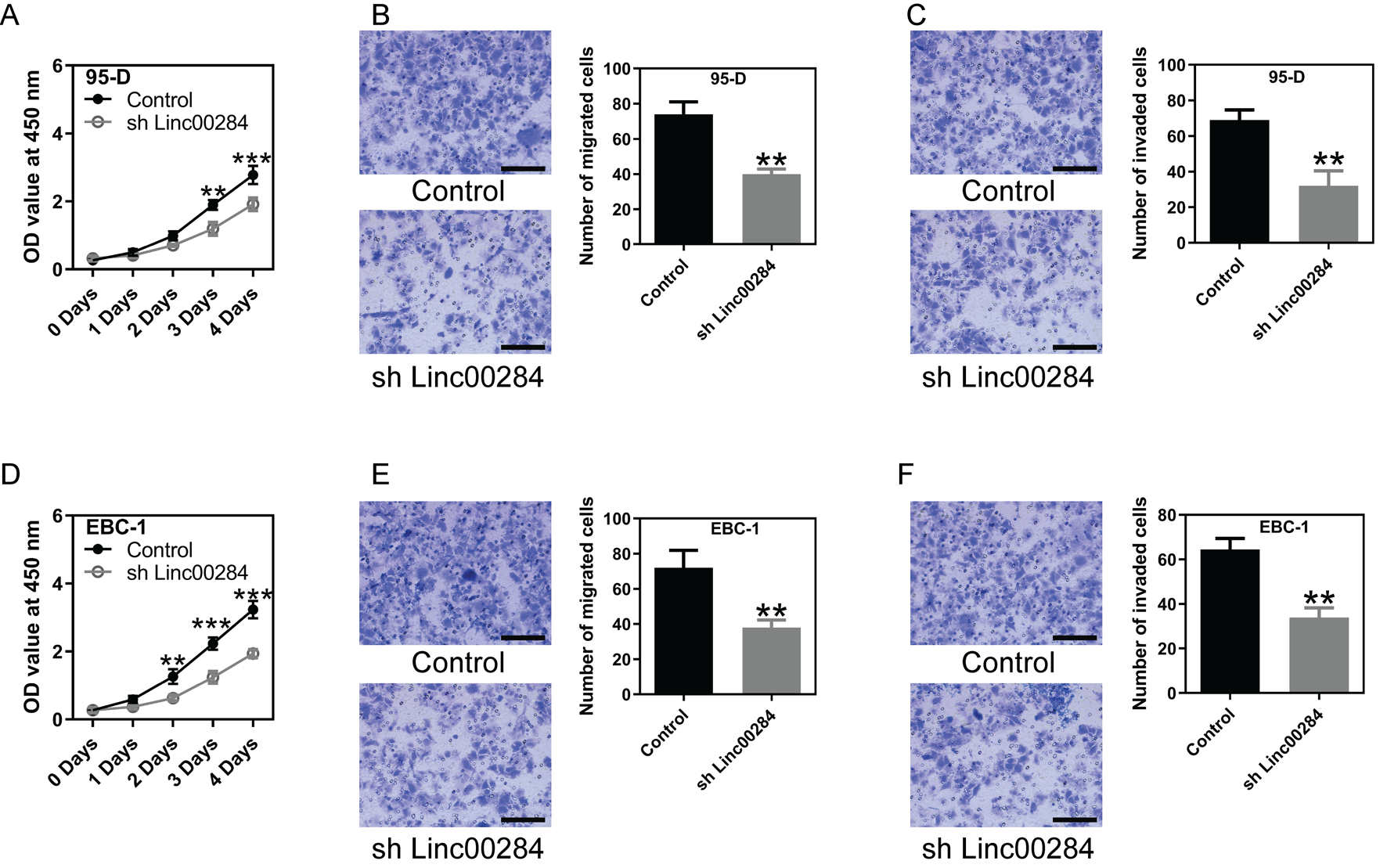

Supplement: Supplementary Figure 2 — Effects of Linc00284 knockdown on proliferation, migration, and invasiveness of human lung squamous carcinoma cells. (A–C) Linc00284 knockdown suppressed the (A) proliferation, (B) migration, and (C) invasion of 95-D human lung squamous carcinoma cells. (D–F) Linc00284 knockdown inhibited the (D) proliferation, (E) migration, and (F) invasion of EBC-1 human lung squamous carcinoma cells. Data were analyzed using unpaired t-test. Error bar represents standard deviation (SD) for triplicate independent experiments. ∗∗P < 0.01 and ∗∗∗P < 0.001, compared with matched control cells. [file Image_2.TIF]
